# Curcumin and Resveratrol as Dual Modulators of the STAT3 Pathway in Lung Cancer: A Comprehensive Review

**DOI:** 10.1002/fsn3.70829

**Published:** 2025-08-25

**Authors:** Mohammad Yasin Zamanian, Klavdiya A. Turkadze, Mehraveh Sadeghi Ivraghi, Maryam Golmohammadi, Maryam Sharifi, Zahra Keshtpour Amlashi

**Affiliations:** ^1^ Department of Physiology, School of Medicine Hamadan University of Medical Sciences Hamadan Iran; ^2^ Department of Pharmacology and Toxicology, School of Pharmacy Hamadan University of Medical Sciences Hamadan Iran; ^3^ Department of Infectious Diseases of Institute of Public Health Named After F.F. Erisman I.M. Sechenov First Moscow State Medical University of the Russian Federation (Sechenov University) Moscow Russia; ^4^ School of Medicine Qazvin University of Medical Sciences Qazvin Iran; ^5^ School of Medicine Shahid Beheshti University of Medical Sciences Tehran Iran; ^6^ MPH, American International University Bangladesh (AIUB) Dhaka Bangladesh; ^7^ Cancer Research Center, Institute of Cancer, Avicenna Health Research Institute Hamadan University of Medical Sciences Hamadan Iran

**Keywords:** curcumin, JAK2/STAT3 signaling pathway, lung cancer, resveratrol, STAT3

## Abstract

Lung cancer, primarily consisting of nonsmall cell lung cancer (NSCLC) and small cell lung cancer (SCLC), remains a significant health challenge despite advancements in treatment. This comprehensive review investigates the therapeutic potential of natural compounds curcumin (CUR) and resveratrol (RES) in targeting the STAT3 signaling pathway, which plays a crucial role in lung cancer progression and metastasis. Specifically, CUR inhibits STAT3 phosphorylation and activation in lung cancer cells, leading to a 40%–60% reduction in tumor size and a significant decrease in the expression of STAT3 target genes such as cyclin D1, VEGF, MMP2, and MMP9. RES demonstrates similar effects by suppressing STAT3 signaling, resulting in a 30%–50% reduction in tumor growth and a marked decrease in the M2 polarization of tumor‐associated macrophages, thereby disrupting the communication between cancer cells and the tumor microenvironment. CUR analogues, such as L48H37 and compound 5 k, also exhibit anticancer effects by blocking the STAT3 pathway. L48H37 suppresses the motility, migration, and invasion of human osteosarcoma cells by inhibiting the JAK/STAT pathway and urokinase plasminogen activator (uPA) expression. Compound 5 k inhibits NSCLC cell growth by regulating the NF‐κB/STAT3 signaling pathways. RES inhibits STAT3 activation and downstream signaling in NSCLC cells, reducing cell migration and invasion while increasing apoptosis by 20%–30%. In vivo studies show that RES can suppress tumor growth by 40%–50% by inhibiting the STAT3/HIF‐1α/VEGF axis. RES also shows promise in overcoming drug resistance in SCLC by inhibiting the STAT3/VEGF pathway and P‐glycoprotein function, potentially resensitizing resistant cells to chemotherapy. These findings underscore the potential of CUR and RES as promising therapeutic agents against lung cancer by targeting the STAT3 signaling pathway and related processes such as angiogenesis, metastasis, and drug resistance. Further research is needed to optimize their bioavailability, understand their molecular mechanisms, and assess their clinical efficacy in combination with standard therapies.

## Graphical abstract

The STAT3 pathway plays a crucial role in lung cancer by regulating cell proliferation, survival, angiogenesis, invasion, and metastasis. CUR and RES have emerged as potential therapeutic agents due to their ability to inhibit the STAT3 pathway, reducing tumor growth, angiogenesis, migration, and invasion.

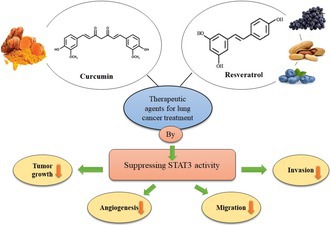

**Abbreviations**
CD105: cluster of differentiation 105
CD31: cluster of differentiation 31
EGFR: epidermal growth factor receptor
FGFR: fibroblast growth factor receptor
GHET1: gastric carcinoma proliferation enhancing transcript 1
HIF‐1α: hypoxia‐inducible factor 1‐alpha
HOTAIR: HOX transcript antisense RNA
ICAM‐1: intercellular adhesion molecule 1
IGFR: insulin‐like growth factor receptor
IL‐10R: interleukin‐10 receptor
IL‐6: interleukin‐6
MALAT1: metastasis associated lung adenocarcinoma transcript 1
MMP2: matrix metalloproteinase‐2
MMP9: matrix metalloproteinase‐9
NF‐ĸB: nuclear factor kappa B
PD‐1: programmed cell death protein 1
PD‐L1: programmed cell death ligand 1
PIAS: protein inhibitor of activated STAT3
PTPs: protein tyrosine phosphatases
SHP‐1: SH2 domain‐containing protein tyrosine phosphatase 1
SOCS: suppressor of cytokine signaling
VEGF: vascular endothelial growth factor

## 1 Introduction

Lung cancer is a significant public health concern due to its high mortality rate. The incidence of new lung cancer cases is substantially higher compared to other cancer types (Sung et al. 2021). The age‐standardized incidence rate (ASIR) of lung cancer varies considerably across countries, ranging from 36.8 per 100,000 in Denmark to 5.9 per 100,000 in Mexico, with men generally having twice the incidence rate compared to women (Li et al. 2023). Despite overall declining trends in many developed countries, lung cancer incidence is projected to reach 3.8 million annual cases globally by 2050, largely due to population growth and aging, as well as increasing tobacco use and air pollution in low‐ and middle‐income countries (Sharma 2022).

Smoking is the primary etiological factor for lung cancer, accounting for approximately 80% of deaths associated with this disease. Other risk factors include exposure to radon and asbestos, as well as prolonged and cumulative exposure to air pollution, particularly emissions containing polycyclic aromatic hydrocarbons (PAHs). Additionally, a personal or family history of lung cancer further increases the risk associated with this disease (Brüske‐Hohlfeld 2009; Quazi and Malik 2022).

According to the World Health Organization (WHO), lung tumors are divided into two main types. Nonsmall cell lung cancer (NSCLC) accounts for 80%–85% of all lung cancer cases, while small cell lung cancer (SCLC) makes up the remaining 15% (Li, Yuan, et al. 2016; Molina et al. 2008; Travis et al. 2015). Current research indicates that more effective methods need to be developed to either cure or manage advanced‐stage lung cancers. This is necessary even with existing anticancer strategies such as surgery, chemotherapy, and radiation used for treating NSCLC and SCLC. Ongoing efforts are crucial to overcoming the challenges posed by advanced lung cancer and improving patient outcomes (Alexander et al. 2020; Yasumoto et al. 2009). Despite significant medical advancements, resistance to important anticancer drugs, such as Paclitaxel (PTX), leads to treatment failure and recurrence of NSCLC (Sosa Iglesias et al. 2018; Zhu and Chen 2019), presenting significant clinical challenges (Cui et al. 2020). Immunotherapy, particularly immune checkpoint blockade (ICB), represents a new therapeutic approach with the potential to improve overall survival outcomes for individuals with lung cancer (Zhou, Qiao, and Zhou 2021). The urgent need for alternative strategies to enhance existing treatments or introduce new ones has become increasingly clear. Clinicians prioritize treatment goals to alleviate pain and improve the quality of life. In recent years, numerous plant extracts have been recognized as potential therapies for lung cancer, gaining greater increasing acceptance from patients. Curcumin (CUR), a polyphenolic compound derived from turmeric root, possesses various pharmacological properties, including antioxidant, anti‐inflammatory, and anticancer effects. These characteristics make it a promising candidate for treating cancer, neurodegenerative diseases, and cardiovascular diseases (Dehzad et al. 2023; Peng et al. 2021; Wong et al. 2021; Lo Cascio et al. 2021; Cox et al. 2022). Other studies have shown that CUR inhibits cancer cell proliferation, metastasis, and promotes apoptosis, supporting its potential effectiveness as an anticancer agent (Man et al. 2018; Wang et al. 2018).

Resveratrol (RES), a polyphenolic stilbene derivative found in grapes, red wine, and other plant sources, has garnered significant interest due to its antineoplastic properties (Ko et al. 2017; Robertson et al. 2022). It exhibits a wide range of cellular and molecular effects, making it a promising candidate for the prevention and treatment of various cancers, including breast, prostate, lung, pancreatic, liver, and colorectal cancers, among others. Furthermore, it demonstrates antioxidant, anti‐inflammatory, and neuroprotective properties (Meng et al. 2020; Honari et al. 2019; Kundu and Surh 2008; Yousef, Vlachogiannis, and Tsiani 2017). The interactions between RES and cellular signaling pathways involved in apoptosis, cell cycle regulation, inflammation, angiogenesis, and metastasis demonstrate its anticarcinogenic properties (Ko et al. 2017; Athar et al. 2009). For example, it inhibits the expression of oncogenes, activates tumor growth‐limiting genes, and regulates the functioning of transcription factors (Xu, Deng, et al. 2021; Zhang et al. 2022).

The majority of NSCLC patients exhibit abnormally high levels of signal transducer and activator of transcription 3 (STAT3) overexpression, which is an essential component of the JAK–STAT pathway (Mohrherr et al. 2020). STAT3 can be phosphorylated by various inflammatory mediators and subsequently translocate to the nucleus, where it regulates the transcription of specific genes (Johnson et al. 2018).

Overactivation of STAT3 is also attributed to the presence of cancer stem cells (CSCs) or tumor‐initiating cells (TICs), which contribute to drug resistance, tumor metastasis, and recurrence (Prasetyanti and Medema 2017). Both CUR and RES have been found to suppress STAT3 activity, suggesting their potential as therapeutic agents against lung cancer (Yousef, Vlachogiannis, and Tsiani 2017; Wan Mohd Tajuddin et al. 2019; Ashrafizadeh, Najafi, et al. 2020). Yang et al. noted that CUR can inhibit IL‐6‐inducible STAT3 phosphorylation in a dose‐ and time‐dependent manner, further suggesting its potential as a therapeutic agent for targeting the IL‐6/JAK/STAT3 pathway in treating SCLC (Yang et al. 2012). Another study showed that CUR can reduce tumors by blocking the JAK2/STAT3 signaling pathway, indicating its potential for lung cancer treatment (Wu et al. 2015). Additionally, RES exerts anticancer effects by inhibiting STAT3 signaling, further indicating its potential as a therapeutic agent for NSCLC (Li, Wang, et al. 2016).

It is crucial to emphasize that CUR and RES are not proposed as replacements for established anticancer drugs, but rather as complementary agents that could potentially enhance treatment efficacy, reduce side effects, and help address the challenge of drug resistance. The aim of this comprehensive review is to elucidate the therapeutic potential of CUR and RES as dual modulators of the STAT3 pathway in lung cancer. By synthesizing current evidence, we seek to provide insights into the molecular mechanisms, anticancer effects, and future directions for leveraging these natural compounds in lung cancer prevention and treatment strategies.

## 2 Methodology

The methodology consisted of several key steps, beginning with a thorough literature search conducted using multiple electronic databases, including PubMed, Scopus, Google Scholar, ScienceDirect, and the Cochrane Library. The search strategy utilized a combination of appropriate keywords and Medical Subject Headings (MeSH) terms, such as “Curcumin,” “Resveratrol,” “Lung cancer,” “Nonsmall cell lung cancer,” “Small cell lung cancer,” “STAT3,” “Signal Transducer and Activator of Transcription 3,” and “JAK/STAT pathway.” Inclusion criteria for the review encompassed original research articles published in peer‐reviewed journals, studies focusing on curcumin or resveratrol in lung cancer, investigations examining the effects on STAT3 signaling, in vitro, in vivo, and clinical studies, and articles published in English. Exclusion criteria included studies not directly related to lung cancer or STAT3 signaling, conference abstracts, case reports, opinion pieces, and articles not available in full text.

## 3 Overview of Current Lung Cancer Treatments

Lung cancer is the leading cause of oncological mortality globally, with NSCLC accounting for 85% of all diagnosed cases (Petrella et al. 2023). Stage III NSCLC is a heterogeneous group characterized by varying tumor volumes, local diffusion, and lymph node involvement, requiring multimodal treatment approaches (Petrella et al. 2023). The primary treatment modalities include surgery, radiotherapy, and systemic therapies, with radical surgery indicated for hilar or single‐station mediastinal lymph node involvement, potentially following neoadjuvant chemotherapy (Petrella et al. 2023).

Surgery remains a crucial component in the multimodal treatment approach for stage III NSCLC, particularly for cases with hilar or single‐station mediastinal lymph node involvement (Petrella et al. 2023).

Radical surgery is often indicated after neoadjuvant chemotherapy to improve resectability and outcomes. However, the optimal treatment strategy for multistation mediastinal lymph node involvement is still debated, with ongoing research exploring the role of surgery in this setting (Saw et al. 2021; Sinn et al. 2022). Advancements in imaging techniques and surgical approaches have aided in the resection of early‐stage lesions, potentially improving outcomes for operable cases (Yang et al. 2017).

However, the optimal treatment for multistation mediastinal lymph node involvement remains debated (Petrella et al. 2023). For brain metastases, a common complication in lung cancer, modern stereotactic radiotherapy combined with targeted therapies is changing the therapeutic landscape (Levis et al. 2023).

Targeted therapies, such as tyrosine kinase inhibitors (TKIs), have revolutionized the management of NSCLC by targeting specific genetic mutations, resulting in improved progression‐free survival and personalized treatment approaches (Kaur et al. 2024). However, the development of drug resistance remains a significant challenge, necessitating the exploration of new strategies to overcome this obstacle.

Chemotherapy continues to be a fundamental aspect of lung cancer management, especially in the case of NSCLC. The use of cytotoxic chemotherapy, whether administered as a monotherapy or in conjunction with other therapeutic approaches such as targeted therapies or immunotherapy, has shown significant clinical advantages in enhancing both survival rates and quality of life for patients diagnosed with early‐stage and advanced NSCLC (Lee 2019). Platinum‐based chemotherapy regimens, such as cisplatin or carboplatin combined with drugs like pemetrexed, paclitaxel, or nab‐paclitaxel, are commonly used in the neoadjuvant, adjuvant, and metastatic settings (Forde et al. 2022; Gandhi et al. 2018). Chemotherapy is advised as a standard treatment option for patients who have experienced disease progression following the administration of targeted therapies, such as tyrosine kinase inhibitors (TKIs) or immune checkpoint inhibitors (ICIs) (Lee 2019). Combination strategies involving chemotherapy and other treatment modalities, such as immunotherapy (e.g., pembrolizumab plus chemotherapy), are actively being explored to enhance therapeutic efficacy and overcome resistance mechanisms (Gandhi et al. 2018). Neoadjuvant chemotherapy combined with immunotherapy (e.g., nivolumab plus chemotherapy) has shown promising results in improving pathological complete response rates and event‐free survival in resectable NSCLC (Forde et al. 2022). Researchers are investigating new drug delivery systems and combination approaches with photodynamic therapy (PDT) to address drug resistance and enhance the efficacy of chemotherapy in lung cancer treatment (El‐Hussein et al. 2021). Immunotherapy has significantly altered the therapeutic landscape for advanced or metastatic NSCLC owing to its sustained effectiveness and tolerable side effects (Xu et al. 2023).

ICIs like anti‐PD‐1 (nivolumab, pembrolizumab) and anti‐PD‐L1 (atezolizumab, durvalumab) have become cornerstones in NSCLC treatment, significantly improving patient survival and quality of life (Shen et al. 2023). Anti‐CTLA‐4 inhibitors (ipilimumab) are also being utilized, often in combination with anti‐PD‐1/PD‐L1 agents (Shen et al. 2023; Roque et al. 2023).

While targeted therapies and immunotherapy have revolutionized lung cancer treatment, chemotherapy remains a fundamental component, particularly in combination strategies, and continues to expand its clinical implications in the era of precision medicine.

Polyphenols, which are compounds naturally found in plants, have demonstrated significant potential as chemopreventive and therapeutic agents for lung cancer (Suvarna et al. 2018). Studies have indicated a negative correlation between the consumption of fruits and vegetables rich in polyphenols and the risk of lung cancer, particularly in high‐risk populations (Amararathna et al. 2016; Wang, Yang, et al. 2019). These polyphenols possess antioxidant, anti‐inflammatory, and antimutagenic properties, effectively safeguarding against lung carcinogenesis caused by carcinogens and oxidative stress (Amararathna et al. 2016). In addition, they can modulate the activity of phase I and II metabolic enzymes, promoting the detoxification of carcinogens and preventing DNA damage (Amararathna et al. 2016). In both in vitro and in vivo studies, polyphenols have exhibited anticancer effects against lung cancer cells by inhibiting cell proliferation, inducing apoptosis, and regulating cell cycle progression (Amararathna et al. 2016). Furthermore, they can target various signaling pathways involved in the progression of lung cancer, such as the PI3K/Akt/mTOR pathway, which ultimately restricts tumor growth (Wang, Yuan, et al. 2022). Polyphenols may provide a potential therapeutic strategy by regulating the expression of microRNAs (miRNAs) associated with the initiation and progression of lung cancer (Li, Zhong, et al. 2022). Through various mechanisms, including the modulation of Bcl‐2 family proteins and activation of caspases, polyphenols can trigger apoptosis or programmed cell death in lung cancer cells (Li, Zhong, et al. 2022). Nevertheless, the clinical application of polyphenols is hindered by their limited bioavailability and rapid clearance from the body (Vieira et al. 2023). Nanoengineered delivery systems, such as liposomes, nanoparticles, and micelles, have been developed to enhance the targeted delivery, solubility, and bioavailability of polyphenols, thereby improving their therapeutic efficacy against lung cancer (Das et al. 2022). In preclinical trials involving xenograft animal models, treatment with polyphenol‐loaded delivery systems has resulted in reduced tumor growth (Vieira et al. 2023). Preclinical investigations utilizing xenograft animal models have demonstrated a reduction in tumor growth following the administration of polyphenol‐loaded delivery systems (Vieira et al. 2023). Furthermore, the integration of polyphenol codelivery systems with polyphenol‐drug delivery systems demonstrates potential as a strategy to enhance anticancer efficacy while mitigating the adverse effects associated with chemotherapy (Vieira et al. 2023). While additional research is necessary to thoroughly understand the fundamental mechanisms and clinical applications, polyphenols demonstrate significant promise as chemopreventive and therapeutic agents for lung cancer, whether used alone or in combination with other treatment modalities.

## 4 Overview of STAT3

STAT comprises a group of transcription factors that bind to target gene promoters to regulate gene expression. These factors operate as bridges that carry signals from the cell membrane to the nucleus, initiating transcription of the corresponding gene (Xia et al. 2023; Philips et al. 2022). STAT3, a member of the STAT family, has six domains. The N‐terminal domain is important for DNA attachment and control of nuclear protein transfer and is ubiquitous in the cytoplasm of human cells (Chalikonda et al. 2021). The central DNA‐binding domain plays a crucial role in DNA binding, while the linker domain influences the stability of DNA binding. The alpha‐helical coiled‐coil domain facilitates interactions between STAT3 signaling and other proteins. Notably, the activation of STAT3 hinges on the phosphorylation of its C‐terminal transactivation domain, with phosphorylation occurring at tyrosine 705 and serine 727 (Chen et al. 2019; Zhao et al. 2022).

The original detection of STAT3 was in IL‐6‐stimulated hepatocytes, which revealed a DNA‐binding activity with a preferential interaction with the acute‐phase response element (Xu, Lin, et al. 2021; Song et al. 2023). STAT3 may be triggered by multiple growth factors, cytokines, and hormones that bind to diverse receptors, including cytokine receptors such as IL‐6R and IL‐10R, and growth factor receptors including EGFR, IGFR, and FGFR (Qin et al. 2019; Liang et al. 2016). Ligand interaction causes the receptor to dimerize, which subsequently recruits JAKs and glycoprotein 130 (gp130), leading to phosphorylation and JAK activation (Garbers et al. 2015). Tyrosine residues on the cytoplasmic side of JAKs are phosphorylated during activation, facilitating the interaction of the SH2 domain associated with STAT3 and the receptors, ultimately resulting in JAK‐mediated phosphorylation of STAT3 (Johnson et al. 2018).

STAT3 phosphorylation and activation depend on receptors, but can also be induced by tyrosine kinases like Abl and c‐SRC (El‐Tanani et al. 2022; Panagi and Thurston 2023). When STAT3 is phosphorylated, it forms homodimers, moves from the cytoplasm to the nucleus, and interacts with coactivators such as apurinic/apyrimidinic endonuclease‐1 (APE) and CREB‐binding protein (CBP) to bind to specific DNA sequences and increase the transcription of target genes (Yu et al. 2014; Guanizo et al. 2018). Normal cells use endogenous inhibitors like PIAS, PTPs, and SOCS to control STAT3 activity (Morris et al. 2018).

The ability of STAT3 to influence the expression of multiple genes allows it to regulate important biological processes including apoptosis, cell death, differentiation, and migration. Abnormal expression of the STAT3 protein has been associated with various malignancies, particularly cancer. Studies have shown that increased STAT3 expression contributes to tumor growth and metastasis. Furthermore, activation of oncogenic STAT3 signaling is linked to drug resistance and resistance to radiation. Therefore, targeting STAT3 signaling is crucial for suppressing tumor progression (To et al. 2022; Ashrafizadeh, Zarrabi, et al. 2020; Li, Yin, et al. 2022; Sadrkhanloo et al. 2023).

Overexpression of activated STAT3 is associated with an unfavorable prognosis in different types of cancer. Continuous activation of STAT3 is mainly a result of hyperactive growth factor receptor tyrosine kinase and increased interactions between stimulatory receptors and ligands. When tyrosine 705 residue is phosphorylated, it allows STAT3 to move into the nucleus and subsequently activate genes targeted by STAT3 (Wang et al. 2004).

**FIGURE 1:**
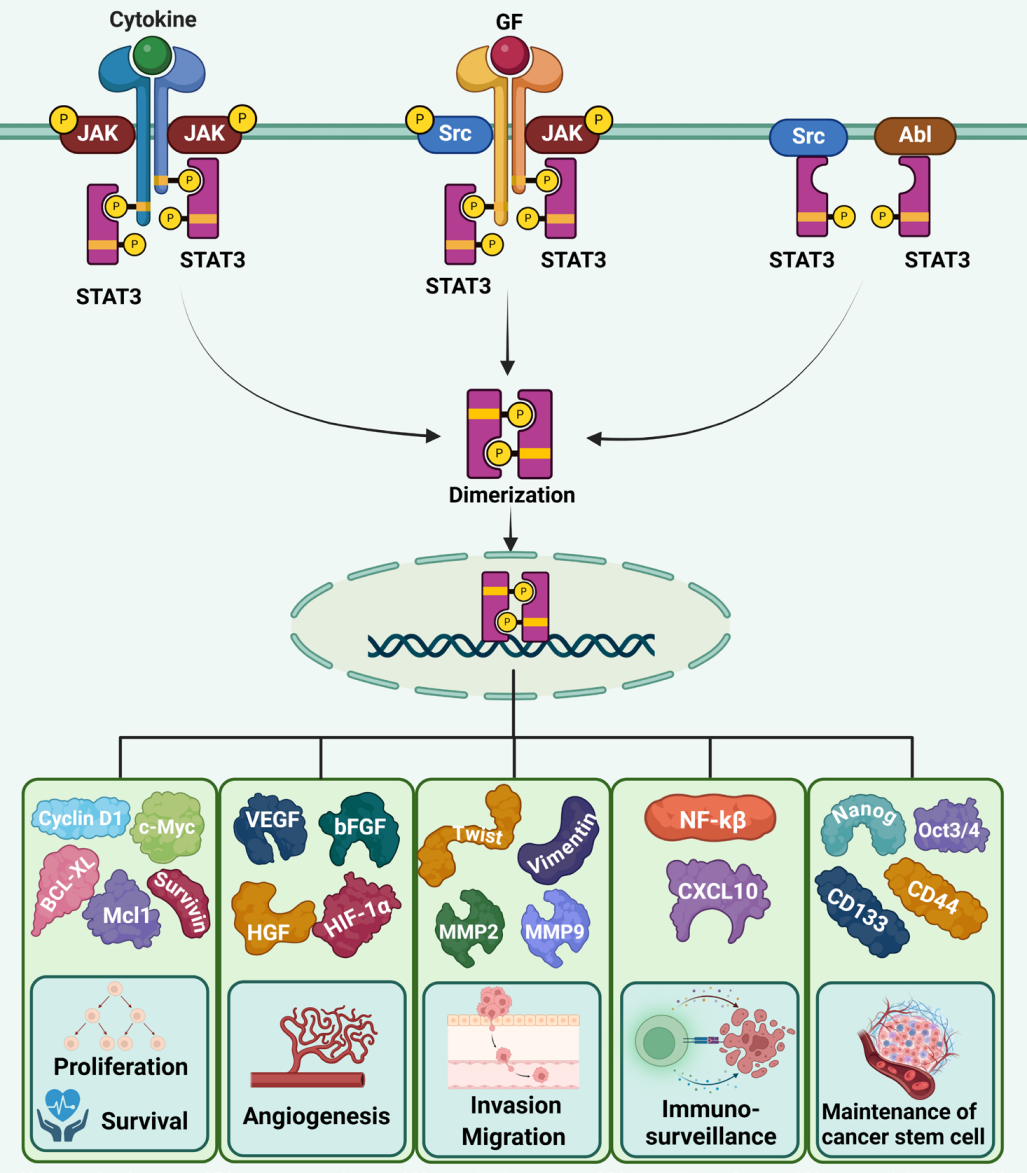
STAT3 signaling is initiated when various ligands bind to their receptors on the cell surface, resulting in the phosphorylation of STAT3. Additionally, nonreceptor tyrosine kinases Src and Abl also directly phosphorylate STAT3. The phosphorylated STAT3 then forms homodimers and translocates to the nucleus. Once in the nucleus, STAT3 regulates gene expression in cellular proliferation and survival, such as CyclinD1, c‐Myc, Survivin, Bcl‐XL, and Mcl1. Moreover, STAT3 upregulates the expression of VEGF, bFGF, HGF, and HIF1α, which participate in processes related to cancer progression. Furthermore, STAT3 promotes invasion and migration by regulating MMP2, MMP9, Twist, and Vimentin expression. It also downregulates immune surveillance by promoting the secretion of chemokines like CXCL10. In addition, STAT3 plays a role in maintaining cancer stem cell properties by regulating the expression of Oct3/4, Nanog, CD133, and CD44.

As an oncogene, STAT3 plays a vital role in various cellular processes such as proliferation, survival, angiogenesis, metastasis, invasion, and immune evasion (Yu et al. 2009; Kim et al. 2016; Bromberg and Darnell 2000; Mohan et al. 2022) (Figure 1).

The constitutive activation of STAT3 has been observed in numerous types of cancers, such as breast, colon, pancreatic, lung, and ovarian cancers, as well as hematological malignancies like lymphomas and leukemias (Molenda et al. 2023; Hashemi et al. 2023; Singh et al. 2023). In cancer cells, the persistent activation of STAT3 contributes to uncontrolled cell proliferation, inhibition of apoptosis, promotion of angiogenesis, and metastasis (Valle‐Mendiola and Soto‐Cruz 2020). STAT3 also plays a role in metabolic reprogramming, facilitating glycolysis and oxidative phosphorylation, which are crucial for tumor cell survival and proliferation (Poli and Camporeale 2015; Tošić and Frank 2021).

The STAT3 pathway plays a crucial role in gastric cancer progression, promoting cell proliferation, survival, angiogenesis, and metastasis. STAT3 is frequently overactivated in gastric cancer cells, contributing to tumor growth and drug resistance (Ashrafizadeh, Zarrabi, et al. 2020; Ashrafizadeh et al. 2023). Noncoding RNAs, including microRNAs and long noncoding RNAs, have emerged as important regulators of the STAT3 signaling pathway in gastrointestinal cancers. These noncoding RNAs can either activate or inhibit STAT3 signaling, thereby influencing cancer cell behavior and treatment outcomes. MicroRNAs such as miR‐124, miR‐223, and miR‐506 have been shown to directly target STAT3 or its upstream activators, suppressing gastric cancer progression. Long noncoding RNAs like HOTAIR, MALAT1, and GHET1 can modulate STAT3 activity through various mechanisms, including acting as competing endogenous RNAs or interacting with STAT3‐related proteins. The complex interplay between noncoding RNAs and the STAT3 pathway contributes to cancer cell proliferation, metastasis, and therapeutic resistance in gastric and other gastrointestinal cancers (Ashrafizadeh et al. 2023, 2021; Yu et al. 2024).

Understanding these regulatory networks provides new insights into cancer biology and offers potential targets for developing novel therapeutic strategies. Targeting the STAT3 pathway and its associated noncoding RNAs may hold promise for improving treatment outcomes in gastric cancer and other gastrointestinal malignancies.

STAT3 activation has been linked to the development of acquired drug resistance in various cancers, including triple‐negative breast cancer (TNBC). By inhibiting STAT3 through the circKIF4A‐miR‐637 axis, it may be possible to resensitize cancer cells to existing therapies, such as chemotherapy, targeted therapy, and immunotherapy (Wu et al. 2024).

Furthermore, STAT3 has been linked to the development of acquired drug resistance, which poses a significant challenge in cancer treatment. Evidence suggests that at least 24 distinct antineoplastic agents, including chemotherapeutic drugs, targeted kinase inhibitors, antihormonal therapies, and monoclonal antibodies, can induce resistance through the STAT3 signaling pathway (Singh et al. 2023). Given the critical role of STAT3 in cancer development and progression, targeting this pathway has emerged as a promising therapeutic strategy. Various approaches have been explored, including small molecule inhibitors, oligonucleotide‐based therapeutics (e.g., siRNA, shRNA, ASO, and ODN‐decoy), and natural compounds (Molenda et al. 2023).

Natural compounds, such as CUR and epigallocatechin gallate, have been shown to inhibit STAT3 and its related signaling pathways, thereby hindering tumorigenesis in several types of cancer, including gastric, pancreatic, and lung cancers (Ham et al. 2022; Zhu et al. 2011; Duan et al. 2016; Mukherjee and Khuda‐Bukhsh 2015).

In summary, STAT3 plays a pivotal role in cancer development, progression, and acquired drug resistance, making it an attractive therapeutic target. Ongoing research efforts are focused on developing effective STAT3 inhibitors, either alone or in combination with existing anticancer therapies, to overcome drug resistance and improve patient outcomes.

Targeting STAT3 and its metabolic pathways represents a promising strategy for cancer treatment, with several inhibitors currently under clinical investigation. TTI‐101 (formerly C188‐9) represents a promising therapeutic approach to targeting STAT3 in advanced tumors. The ongoing clinical trial NCT03195699 is expected to provide valuable insights into the potential of this phosphorylation inhibitor as a treatment option for a wide range of cancer types (breast cancer, NSCLC, hepatocellular carcinoma and colorectal cancer). This compound is designed to inhibit the phosphorylation of STAT3, a critical step in its activation and subsequent role in promoting cancer cell growth and survival.

SC‐43 is a promising therapeutic agent for the treatment of NSCLC and biliary tract cancer. The ongoing clinical trial NCT04733521 is expected to provide valuable insights into its safety and efficacy, paving the way for further clinical investigations and potential approval for clinical use. SHP‐1‐mediated inhibitor of STAT3 phosphorylation. SC‐43 works by inhibiting the phosphorylation of STAT3, a key step in its activation. This inhibition is mediated through the activation of SHP‐1, a protein tyrosine phosphatase that dephosphorylates STAT3, thereby preventing its activation and subsequent oncogenic effects (Tošić and Frank 2021).

AZD9150 is a Generation 2.5 antisense oligonucleotide (ASO) designed to reduce the expression of STAT3 protein by down‐regulating STAT3 messenger RNA (Mccoon et al. 2015; Xu et al. 2019).

STAT3 is involved in tumor growth and immune evasion. Durvalumab, on the other hand, is a PD‐L1 inhibitor that blocks the interaction between PD‐L1 and PD‐1, allowing T cells to recognize and kill tumor cells (Chen et al. 2023; Liu et al. 2021).

The clinical trial NCT03421353 is a phase Ib/II, open‐label multicentre study designed to evaluate the safety, tolerability, pharmacokinetics, and preliminary antitumor activity of AZD9150 in combination with durvalumab, with or without chemotherapy, in patients with advanced solid tumors and NSCLC. The clinical trial NCT03421353 represents an important step in the development of combination therapies for cancer treatment. By targeting the STAT3 pathway, this approach may offer new treatment options for patients with advanced solid tumors and NSCLC, potentially overcoming the limitations of current therapies (Tošić and Frank 2021).

Napabucasin, also known as BBI608, is a phosphorylation inhibitor currently being investigated for the treatment of NSCLC in clinical trials (Hubbard and Grothey 2017).

The clinical trial (NCT02826161) aims to evaluate the safety, tolerability, pharmacokinetics, and efficacy of napabucasin in patients with NSCLC. Given the critical role of STAT3 in cancer pathogenesis, inhibiting its phosphorylation represents a promising therapeutic approach (Tošić and Frank 2021).

The clinical trial (NCT02983578) is testing the safety, tolerability, pharmacokinetics, and efficacy of IONIS‐STAT3Rx (AZD9150) in patients with advanced, refractory pancreatic, NSCLC, and colorectal cancer. This drug targets STAT3, offering a new cancer therapy approach by disrupting signaling pathways that promote cancer cell growth, survival, and spread (Tošić and Frank 2021).

## 5 The Application of Nanoparticles in the Treatment of Lung Cancer

Nanoparticles have emerged as a promising tool in the treatment of lung cancer, offering targeted drug delivery and enhanced therapeutic efficacy (Wang, Zhou, et al. 2022; Holder et al. 2023). Recent advancements in nanotechnology have led to the development of various nanocarrier systems, including organic, inorganic, and hybrid nanoparticles, each with unique advantages for lung cancer therapy (Girigoswami and Girigoswami 2023; Mottaghitalab et al. 2019). These nanocarriers can overcome the limitations of conventional chemotherapy by improving drug solubility, increasing tumor penetration, and reducing systemic toxicity (Holder et al. 2023; Silva et al. 2024).

Notably, hybrid nanoparticles combining organic and inorganic components have demonstrated improved diagnostic and therapeutic outcomes compared to conventional nanocarriers, offering the ability to incorporate multiple drugs, targeting agents, and photosensitive reagents for more sensitive and specific lung cancer treatment (Mottaghitalab et al. 2019; Woodman et al. 2021). Among the various nanoparticle types, chitosan‐based nanoparticles have gained attention due to their unique biological properties, including antitumor activity and mucoadhesive capacity, which can enhance drug efficacy when administered via the pulmonary route (Silva et al. 2024).

Furthermore, nanoparticle‐based systems have shown promise in overcoming drug resistance and improving the efficacy of radiotherapy in lung cancer treatment, potentially leading to better patient outcomes.

## 6 Curcumin Modulates the STAT3 Signaling Pathway to Inhibit Lung Cancer

CUR is a hydrophobic, low‐molecular‐weight polyphenolic molecule with a bright yellow color isolated from the rhizome of turmeric (Flanders and Gramlich 2022). It may participate in a variety of physical, chemical, and biological processes due to its unique chemical structure. Its chemical name, diferuloylmethane (1,7‐bis‐(4‐hydroxy‐3‐methoxyphenyl)‐1,6‐heptadiene‐3,5‐dione), reflects its composition as a bis‐α, β‐unsaturated β‐diketone, formed by the coupling of two ferulic acid molecules with a methylene bridge (E Wright et al. 2013; Liang et al. 2009).

Based on the Food and Drug Administration (FDA) documents provided, CUR has been determined to be generally recognized as safe (GRAS) for use as a food ingredient in certain categories and at specified maximum levels (U.S. Food and Drug Administration 2018).

Tremendous progress has been made in understanding the molecular biology of cancer, yet it remains undefeated (Surh and Chun 2007). CUR is a highly versatile molecule that can be used in cancer treatment as both a chemopreventive agent and as part of chemotherapy, with minimal side effects (Allegra et al. 2017). For example, it can interfere with multiple stages of carcinogenesis (Kabir et al. 2021), and is easily accessible, cost‐effective, and safe (George et al. 2021; Park et al. 2013; Lelli et al. 2017).

The NSCLC cell line H460 (NCI‐H460) is widely used in numerous experiments to explore the effects of various compounds, such as CUR, on lung cancer cells (Abbas Albaayit et al. 2021; Chang and Chen 2015). The NCI‐H460 cell line has played a crucial role in gaining insights into the mechanisms of lung cancer progression, drug resistance, and potential targeting of cancer stem‐like cells (Herreros Pomares 2020).

In a study by Wu et al., CUR significantly inhibited the activity of the JAK2/STAT3 signaling pathway in NCI‐H460 tumor spheroids, resulting in downregulation of p‐JAK2 and p‐STAT3 in a time‐ and dose‐dependent manner. Furthermore, molecular docking analysis showed that CUR can bind to JAK2 to inhibit its activity. Other research has shown that overexpression of STAT3 promotes tumor sphere formation, and its inhibition by CUR halts lung cancer progression (Wu et al. 2015).

The CUR analogue L48H37 impedes the invasion and migration of human osteosarcoma cells by blocking the JAK/STAT pathway and urokinase plasminogen activator (uPA). A previous study revealed that L48H37 successfully suppressed the motility, migration, and invasion of human osteosarcoma U2OS and MG‐63 cells without inducing cytotoxicity. It was also reported that L48H37 reduced the quantity of uPA protein, mRNA expression, and promoter activity, and repressed the JAK/STAT signaling pathway by reducing the phosphorylation of STAT3, JAK1, JAK2, and JAK3 in U2OS cells. The data imply that L48H37 may be a suitable option for antimetastatic therapy of human osteosarcoma (Lu et al. 2020).

STAT3 directly activates the VEGF gene at the transcriptional level, thereby promoting angiogenesis in NSCLC. This process ultimately contributes to the progression of cancer and adversely affects the overall survival of affected patients (Chen and Han 2008; Parakh et al. 2021). In NSCLC, targeting the STAT3/VEGF axis can significantly reduce tumor‐associated VEGF, angiogenesis, and vascular permeability in lung cancer cells, indicating the therapeutic significance of inhibiting STAT3 (Mohrherr et al. 2020; Kang et al. 2019).

CUR ameliorates NSCLC by reducing tumor size and weight in an ectopic xenograft model, leading to a significant decrease in hemoglobin content, as well as mRNA expression of CD31 and CD105 within the tumor tissue, denoting its antiangiogenic potency. Furthermore, CUR suppresses the STAT3 pathway, as evidenced by decreased levels of phosphorylated STAT3, JAK, VEGF, Bcl‐xL, and Cyclin D1 in ectopic xenografts. In vitro assays corroborated these findings and demonstrated not only curcumin's ability to inhibit cell migration but also tube formation in NCI‐H460 cells. Transfecting with a dominant active mutant of STAT3 notably counteracted CUR‐induced inhibitory effects on NCI‐H460 cells, thereby implicating STAT3 as one of the possible mechanisms by which CUR exerts its antiangiogenic effects in cancer cells. These results collectively imply promising potential for CUR as an agent to therapeutically target STAT3 in treating NSCLC (Xu and Zhu 2017).

Minichromosome maintenance complex component 2 (Mcm2) is an essential protein that plays a critical role in the process of DNA replication (Sakwe et al. 2007). It belongs to the MCM protein family, required for the development of the replicative helicase complex essential for DNA replication initiation (Malysa et al. 2024; Sun et al. 2021). Mcm2 works in unwinding the DNA double helix and allowing DNA replication throughout the cell cycle (Lei 2005). Its expression acts as an indication of cellular proliferation, often exploited as a marker for tissue proliferative ability (Yousef, Furrer, et al. 2017).

CUR treatment was shown to cause in vitro dose‐dependent suppression of STAT3 phosphorylation, indicating inhibition of the STAT3 pathway and consequently decreased cellular proliferation. Additionally, the lung tissue of rats administered CUR had lower expression of proliferative markers including Cyclin D1, STAT3, and Mcm2. These findings highlight curcumin's ability to lower STAT3 pathway activity and limit the proliferative capability of lung tissue, demonstrating its chemopreventive efficacy against lung cancer (Alexandrow et al. 2012).

According to prior research, CUR inhibits the production of invasive proteins, including MMP‐2, MMP‐7, and ICAM‐1, as well as proliferative proteins like survivin, bcl‐xl, and cyclin B1. Moreover, it inhibits the production of Cyclin B1, a protein linked to the advancement of the G2/M phase, resulting in G2/M phase cell cycle arrest in SCLC cells. It also suppresses IL‐6‐induced STAT3 phosphorylation in a dose‐ and time‐dependent manner, indicating its potential as a multitarget medication in anticancer treatment (Yang et al. 2012).

Further investigation demonstrated that CUR exhibits dose‐ and time‐dependent inhibition of NCI‐H292 cell growth via the upregulation of apoptosis, as shown by the Annexin V apoptosis test and Caspase‐3/7 activity assessments. Furthermore, CUR increases the expression of suppressors of cytokine signaling proteins, SOCS1 and SOCS3, which act as negative regulators of STAT3. The research also discovered that CUR increases the expression of Forkhead box transcription factor A2 (FOXA2) or hepatocyte nuclear factor 3 beta, a suppressor of tumor metastasis in human lung malignancies. It also blocks the STAT3 signaling pathway. Additionally, the research revealed the possible function of suppressors of cytokine signaling (SOCS1 and SOCS3) proteins in mediating the inhibitory effects of CUR on STAT3 activation and FOXA2 expression (Tang et al. 2018). Overall, the data provide new insights into the molecular processes underlying the anticancer actions of CUR in SCLC cells and highlight FOXA2 and STAT3 as prospective therapeutic targets.

Compound 5 k is a 1,2,3‐triazole CUR derivative that was produced and tested for its anticancer efficacy in the research (Ashrafizadeh et al. 2021). It is a dichloro‐substituted phenyltriazole methyl CUR that has shown remarkable anticancer efficacy by decreasing tumor cell growth and inducing apoptosis both in vitro and in vivo (Ashrafizadeh et al. 2021). Research demonstrates that compound 5 k, a 1,2,3‐triazole CUR derivative, triggers the Mitogen‐Activated Protein Kinase (MAPK) signaling pathway in a time‐dependent manner, reducing A549 cell growth and promoting apoptosis. Additionally, compound 5 k represses the NF‐κB/STAT3 signaling pathway by upregulating the expression of IκBα and lowering the levels of NF‐κB, p‐STAT3, and β‐catenin. These results imply that compound 5 k may possibly treat NSCLC by regulating the NF‐κB/STAT3 signaling pathways and decreasing cancer cell proliferation (Zhi et al. 2022).

According to research, compound 5 k, a 1,2,3‐triazole CUR derivative, triggers the Mitogen‐Activated Protein Kinase (MAPK) signaling pathway in a time‐dependent manner, reducing A549 cell growth and promoting cell death. Additionally, compound 5 k represses the NF‐κB/STAT3 signaling pathway by upregulating the expression of IκBα and lowering the levels of NF‐κB, p‐STAT3, and β‐catenin. These results imply that compound 5 k may possibly treat NSCLC by regulating the NF‐κB/STAT3 signaling pathways and decreasing cancer cell proliferation (Zhi et al. 2022).

**FIGURE 2:**
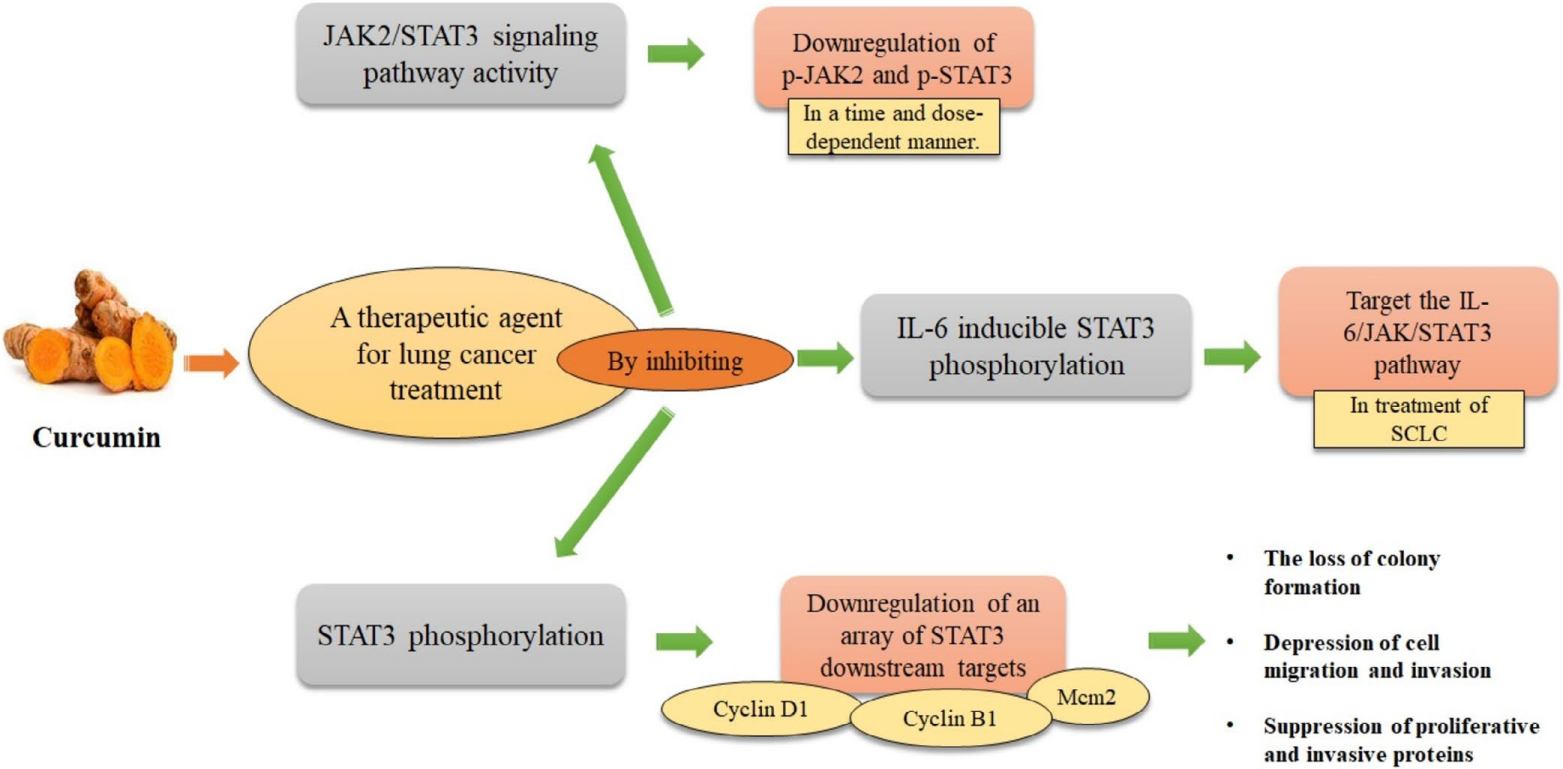
Effects of CUR in treating lung cancer by targeting the STAT3 signaling pathway. This figure shows how CUR acts as a therapeutic agent for lung cancer treatment by inhibiting multiple pathways. Through inhibition, CUR downregulates JAK2/STAT3 signaling pathway activity, leading to reduced phosphorylation of JAK2 and STAT3 proteins in both a time and dose‐dependent manner. Additionally, CUR inhibits IL‐6 inducible STAT3 phosphorylation, which targets the IL‐6/JAK/STAT3 pathway specifically in SCLC treatment. Furthermore, CUR inhibits STAT3 phosphorylation, resulting in downregulation of several STAT3 downstream targets including Cyclin D1, Cyclin B1, and Mcm2.

Table 1 and Figure 2 provide a summary of the effects of CUR on lung cancer through STAT3.

**TABLE 1:** Curcumin effects on STAT3 pathway in lung cancer.

Authors	Dosage of curcumin (CUR)	Type of model	Mechanisms
Wu et al. (2015)	40 mg/kg	Mice	CUR affects the STAT3 pathway in mice by significantly inhibiting tumor growth and downregulating the expression of p‐STAT3 and p‐JAK2 in tumor samples.
Xu and Zhu (2017)	100 mg/kg	Mice	CUR affects the STAT3 pathway in mice by inhibiting its activation, leading to a reduction in tumor growth and angiogenesis. In a mice model of NSCLC, CUR treatment resulted in a significant decrease in tumor size and weight.
Alexandrow et al. (2012)	25 μM	H441 cells	CUR treatment resulted in reduced cell proliferation in a dose‐dependent manner for the H441 cells. These findings indicate that CUR can suppress the activity of the Stat3 pathway in lung cancer‐derived cells, leading to a reduction in cell proliferation.
Yang et al. (2012)	15 μM	NCI‐H446 and NCI‐1688 cells	CUR inhibited the phosphorylation of STAT3 at tyrosine 705 in NCI‐H446 and NCI‐1688 cells. This inhibition of STAT3 phosphorylation occurs in a dose‐dependent and time‐dependent manner. Additionally, CUR treatment suppressed IL‐6‐induced STAT3 phosphorylation in these cells.
Tang et al. (2018)	40 μM	NCI‐H292 cells	CUR treatment reduced the phosphorylation of STAT3 in both the cytoplasmic and nuclear fractions of NCI‐H292 cells. This inhibition of STAT3 signaling was associated with an increase in the expression of FOXA2, a transcription factor that was frequently lost in LSCC tissues. The upregulation of FOXA2 by CUR was found to be mediated by the inhibition of the STAT3 pathway.

Abbreviations: Bcl‐xL, B‐cell lymphoma‐extra large; FOXA2, Forkhead Box A2; IL‐6, interleukin 6; JAK, Janus Kinase; Mcm2, minichromosome maintenance complex component 2; NF‐κB, nuclear factor kappa B; SCLC, small cell lung cancer; SOCS, suppressor of cytokine signaling; STAT3, signal transducer and activator of transcription 3; VEGF, vascular endothelial growth factor.

## 7 Regulation of the STAT3 Signaling Pathway by Resveratrol Inhibits Lung Cancer

Research indicates that animals exhibit a high tolerance for RES even at large dosages (1000–1500 mg/kg/day) (Christenson et al. 2016). However, certain clinical trials report mild‐to‐moderate gastrointestinal symptoms with daily doses between 2.5 and 5 g/Kg and diarrhea when given twice per day at a dose level higher than 2 g/Kg (Juan et al. 2002; Cottart et al. 2010). Notably, micronized RES appears to be better tolerated (Howells et al. 2011). Humans absorb approximately 70% of orally or intravenously administered 14C‐labeled RES (Zhang et al. 2021). Phase II metabolic enzymes, particularly UDP‐glucuronosyltransferases and sulfotransferases in the liver and intestinal microsomes, critically facilitate its metabolism, consequently producing RES glucuronides and sulfates (Bishayee et al. 2010).

A previous study showed that RES exerts concentration‐dependent and time‐dependent cytotoxicity on the NSCLC cell line (A549) by inhibiting proliferation, migration, and invasion, and by promoting apoptosis. Additionally, RES downregulated STAT3 protein expression. This study postulated that inhibiting the STAT3 pathway may prevent metastasis, thus highlighting its therapeutic potential in treating NSCLC (Li, Wang, et al. 2016).

The STAT3/HIF‐1α/VEGF signaling pathway plays significant roles in promoting tumor growth and progression, such as regulating cellular responses to low oxygen levels (hypoxia) and promoting the expression of genes that support tumor growth and angiogenesis (Zimna and Kurpisz 2015).

In a study by Wang et al., RES significantly decreased the expression of STAT3, HIF‐1α, STAT3, and VEGF mRNA and proteins, lung weight index, as well as lung tumor burden levels in a nude rat model of NSCLC. Collectively, these findings suggest that RES may be a potential drug that can be used to inhibit the progression of NSCLC by targeting and inhibiting the STAT3, HIF‐1α, and VEGF signaling pathway (Wang et al. 2020).

Tumor‐associated macrophages (TAMs) are immune cells in the tumor microenvironment, playing a significant role in cancer progression (Wang, Li, et al. 2019). These macrophages are recruited by primary tumors and circulating tumor cells and undergo functional polarization driven by tumor‐derived factors (Christofides et al. 2022). TAMs play a significant role in multiple phases of cancer metastasis, encompassing cancer invasion, intravasation, survival within the circulatory system, and sustained proliferation at secondary tumor sites (Dallavalasa et al. 2021). They are known to promote tumor growth, invasion, and local immunosuppression, and are involved in evading antitumor immune responses (Pan et al. 2020). TAMs are characterized by their plasticity and flexibility, with different activation states denoted as M1 and M2 (Jayasingam et al. 2020). The M2‐like TAMs mainly promote tumor angiogenesis, migration, and invasion while suppressing antitumor immune responses (Pan et al. 2020). The presence of TAMs has been linked to poor lung cancer prognosis, and their polarization status is crucial in determining their tumor‐promoting or tumor‐preventing role (Tariq et al. 2017). Therefore, targeting M2‐like TAMs has been identified as a promising approach for adjuvant anticancer therapies (Han et al. 2021).

Sun et al. reported that RES effectively inhibits the growth of lung cancer by suppressing the protumor activation of TAMs. In vivo experiments using a mouse model showed that RES treatment significantly reduced lung tumor size and weight, and downregulated the activation of STAT3 in tumor cells. Additionally, RES inhibited the infiltration and polarization of TAMs in the tumor tissues. Studies show that RES inhibits the M2 polarization of human monocyte‐derived macrophages (HMDMs) induced by lung cancer cell‐conditioned medium in vitro. Furthermore, RES inhibited lung cancer cell proliferation when cocultured with human macrophages. The study also revealed that RES decreased the secretion of specific cytokines associated with M2 polarization and increased the secretion of cytokines associated with M1 polarization in HMDMs. These findings suggest that RES has the potential to inhibit the progression of lung cancer by disrupting the communication between tumor cells and TAMs, ultimately leading to a reduction in tumor growth and offering a promising avenue for enhancing cancer therapy efficacy (Sun et al. 2017).

P‐glycoprotein (P‐gp) is a protein that is encoded by the MDR1 gene and is classified within the ATP‐binding cassette (ABC) transporter family (Mizutani et al. 2008). It is essential for the development of multidrug resistance (MDR) in cancer cells because it actively drives a variety of substances out of cells (Chen et al. 2016). P‐gp expression levels have been linked to resistance to therapy in several cancers, including SCLC. For instance, when employed against drug‐resistant cells like H69AR overexpressing P‐gp, chemotherapeutic medications like Adriamycin lose their efficacy (Triller et al. 2006; Hou et al. 2021). Therefore, comprehending the function of P‐gp is essential in formulating strategies to counteract medication resistance in cancer therapy.

According to research by Hou et al. the STAT3/VEGF signaling pathway is activated in SCLC cells by the inflammatory milieu, which leads to treatment resistance. On the other hand, research suggests that RES inhibits the STAT3/VEGF pathway and the inflammatory milieu, which in turn helps reduce treatment resistance in SCLC cells. It has been discovered that RES inhibits P‐gp function and lowers the expression levels of inflammatory mediators, which reduces drug resistance in SCLC cells. Furthermore, by addressing drug resistance, it has the potential as a therapeutic agent for the treatment of SCLC. This work emphasizes how the STAT3/VEGF pathway contributes to medication resistance and how RES might help prevent it. These results establish the foundation for more research on RES as a potential therapeutic intervention to address drug resistance in SCLC (Hou et al. 2021).

**FIGURE 3:**
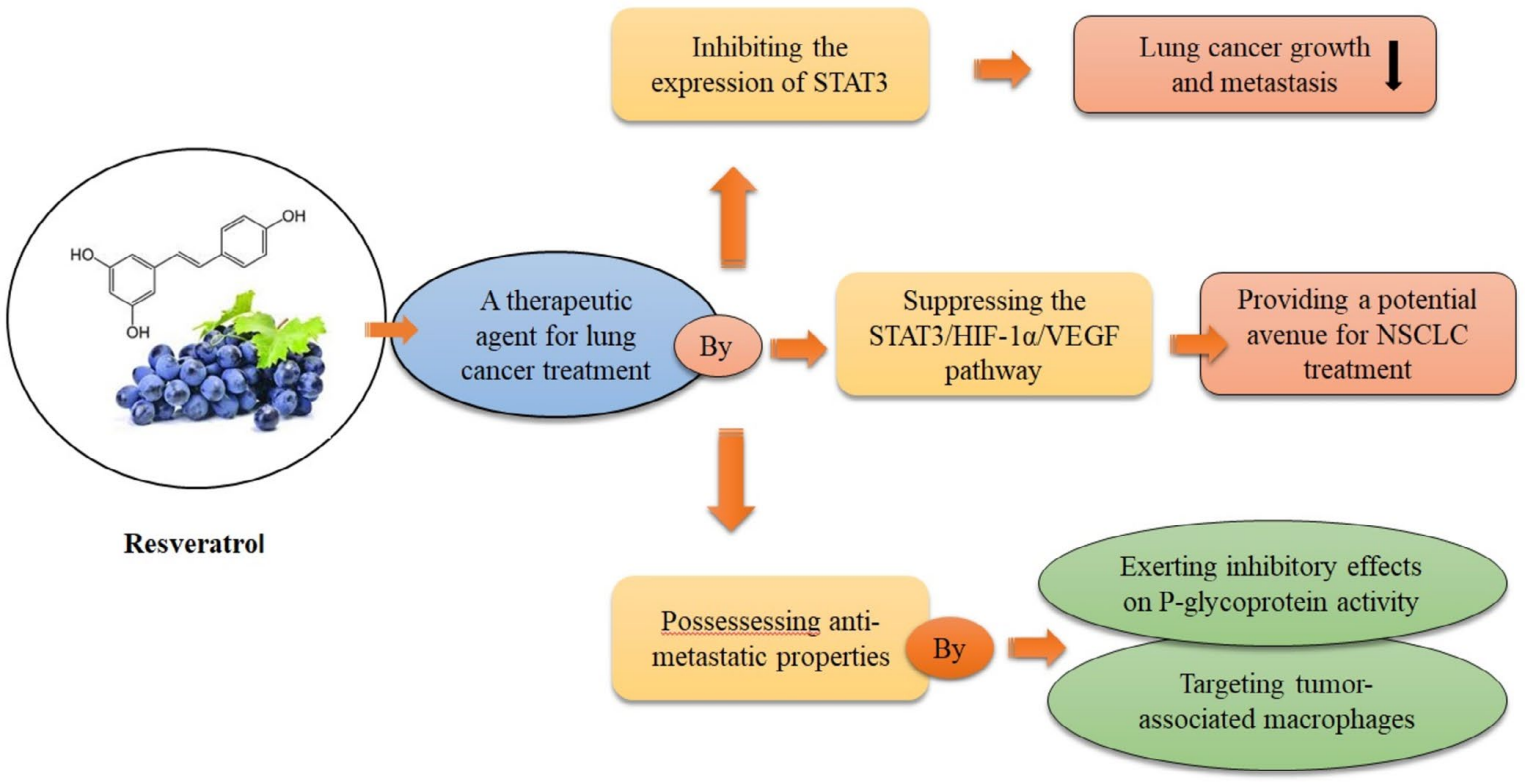
Effects of RES in treating lung cancer by targeting the STAT3 signaling pathway. This figure illustrates how RES acts as a therapeutic agent for lung cancer treatment through multiple pathways. RES inhibits the expression of STAT3, HIF‐1α, and VEGF, leading to reduced lung cancer growth and metastasis. Through suppression of the STAT3/HIF‐1α/VEGF pathway, it provides a potential avenue for treating NSCLC. Additionally, RES possesses antimetastatic properties by exerting inhibitory effects on P‐glycoprotein activity and targeting tumor‐associated macrophages. The compound's ability to suppress the inflammatory microenvironment and modulate multiple signaling pathways makes it a promising therapeutic agent for lung cancer treatment.

Table 2 and Figure 3 show a summary of the effects of RES on lung cancer through STAT3.

**TABLE 2:** Resveratrol effects on STAT3 pathway in lung cancer.

Authors	Dosage of resveratrol (RES)	Type of model	Mechanisms
Li, Wang, et al. (2016)	50 μM	A549 cells	RES exhibited a time‐dependent inhibition on STAT3 mRNA and protein expression in A549 cells. Specifically, the results of real‐time PCR and western blotting showed that the expression of STAT3 mRNA and protein decreased with time, especially at 48 and 72 h after RES administration.
Wang et al. (2020)	50 mg/kg/day for 12 weeks	Rat	RES treatment led to a significant decrease in the mRNA and protein levels of STAT3 in the NSCLC+RES group compared to the NSCLC group. This suggests that RES can inhibit the expression of STAT3 in the rat model of NSCLC. The study also proposed the STAT3/HIF‐1α/VEGF pathway as a potential drug target for developing new chemotherapy agents derived from RES for the treatment of NSCLC.
Sun et al. (2017)	100 mg/kg	Mice	RES treatment led to a significant reduction in tumor growth, which was associated with the inhibition of cell proliferation and decreased expression of p‐STAT3 in tumor tissues. Additionally, RES was found to inhibit F4/80 positive expressing cells and M2 polarization in the tumors. These results suggest that RES effectively inhibits lung cancer progression by suppressing the protumor activation of tumor‐associated macrophages (TAMs) and decreasing the expression of p‐STAT3 in tumor tissues in mice.
Hou et al. (2021)	100 μM	H69 and H69AR	RES suppressed the inflammatory microenvironment, decrease the expressions of p‐NF‐ĸB, IL‐1β, IL‐8, and IL‐23, and inactivate the STAT3/VEGF signaling pathway, thereby reducing drug resistance in lung cancer cells.

Abbreviations: HIF‐1α, hypoxia‐inducible factor 1‐alpha; NSCLC, nonsmall cell lung cancer; P‐gp, P‐glycoprotein; SCLC, small cell lung cancer; STAT3, signal transducer and activator of transcription 3; TAMs, tumor‐associated macrophages; VEGF, vascular endothelial growth factor.

## 8 Future Directions and Limitations

The search results suggest that numerous clinical trials have been conducted to assess the effectiveness of CUR in various types of cancer, including breast cancer, colorectal cancer, and prostate cancer (Talakesh et al. 2022; Howells et al. 2019; Choi et al. 2019). Preclinical studies have demonstrated promising anti‐inflammatory and antiproliferative effects of CUR, supporting its potential as a therapeutic agent for cancer (Xin and Zhang 2024; Ebrahimi et al. 2021). However, the search results do not provide specific information regarding the outcomes of clinical trials involving CUR for lung cancer. Although RES has exhibited various pharmacological activities, including anticancer effects in clinical studies (Zhu et al. 2012; Ostwal et al. 2022), the lack of clinical data for lung cancer suggests that further preclinical studies and early‐phase clinical trials may be necessary before considering larger trials for this disease. In summary, the current status of CUR in lung cancer appears to be more advanced, with some available clinical data, although the extent of this data is unclear from the search results. On the other hand, RES seems to be primarily in the preclinical stage for its applications in lung cancer, and additional preclinical and early‐phase clinical studies may be required before initiating larger trials.

Understanding the ADME (absorption, distribution, metabolism, and excretion) properties of a drug or compound is crucial for determining its pharmacokinetic profile, optimal dosing regimen, potential drug interactions, and overall safety and efficacy in clinical settings (Zhou, Zheng, and McClements 2021; Jaisamut et al. 2017).

One of the main problems in clinical research with polyphenols like CUR and RES is their limited bioavailability. This means that when taken orally, only a small amount of the compound gets absorbed into the bloodstream and reaches the target tissues (Jakobušić Brala et al. 2023). A review study highlights that plant‐derived compounds such as CUR and RES exhibit promising anticancer effects in preclinical studies. However, the challenge lies in their restricted bioavailability, which presents a significant obstacle when attempting to apply these findings in clinical settings (Sukprasansap and Chanvorachote 2022). CUR and RES are rapidly metabolized and eliminated from the body, further reducing their bioavailability and limiting their therapeutic efficacy (Sukprasansap and Chanvorachote 2022; Liu et al. 2019). To overcome the bioavailability issues, the reviews emphasize the need to design new codelivery systems or formulations that can improve the absorption, distribution, and bioavailability of these polyphenols for future clinical research (Jakobušić Brala et al. 2023; Sukprasansap and Chanvorachote 2022; Liu et al. 2019). A new self‐microemulsifying formulation incorporating both CUR and RES was created utilizing Capryol 90 as the oil component, Cremophor EL as the surfactant, and Labrasol as the cosurfactant. This formulation resulted in a tenfold increase in the total plasma concentrations of CUR and a sixfold increase in RES, in comparison to the unformulated combination, following oral administration in rabbits (Jaisamut et al. 2017).

Encapsulating CUR and RES in nanoparticles, such as polymeric nanoparticles, lipid‐based nanoparticles, inorganic nanoparticles, or carbon‐based nanoparticles, can improve their solubility, stability, and bioavailability by protecting them from degradation and enhancing cellular uptake (Vieira et al. 2023; Rassu et al. 2023). CUR, RES, and quercetin were loaded into soybean oil‐in‐water nanoemulsions, and their encapsulation efficiency was relatively high (75%–87%). This encapsulation can improve the solubility and absorption of these lipophilic polyphenols (Zhou, Zheng, and McClements 2021).

To address these gaps and promote the clinical progression of CUR and RES for the treatment of lung cancer, future endeavors should prioritize the implementation of rigorous preclinical investigations, the resolution of bioavailability obstacles, and the initiation of well‐designed clinical trials. The collaboration between academia, industry, and regulatory agencies is imperative to facilitate the translation of these naturally occurring compounds into efficacious therapeutic approaches for individuals diagnosed with lung cancer.

## 9 Future Research and Developments

### 9.1 Enhancing Bioavailability

One of the main challenges in translating the promising preclinical results of CUR and RES to clinical applications is their limited bioavailability. Future research should focus on:

– Developing advanced drug delivery systems to improve the absorption and bioavailability of CUR and RES.
– Exploring nanoformulations, such as polymeric nanoparticles, lipid‐based nanoparticles, and inorganic nanoparticles, to enhance cellular uptake and protect these compounds from rapid metabolism.
– Investigating codelivery systems that combine CUR and RES with other therapeutic agents to potentially synergize their effects and improve overall efficacy.

### 9.2 Clinical Trials

RES shows promising potential for cancer prevention and treatment based on preclinical studies, but more clinical research is needed to establish its efficacy in humans. Several phase I/II clinical trials are currently underway to evaluate resveratrol's effects on various cancers, including colon cancer, lymphoma, and colorectal cancer. These trials aim to assess resveratrol's ability to modulate cancer‐related biomarkers, induce apoptosis, inhibit cell proliferation, and determine its optimal dosing and safety profile in cancer patients. A 10‐year epidemiological study found that women who consumed RES from grapes had a 50% or greater reduction in breast cancer risk, suggesting potential benefits from dietary sources. However, the low bioavailability of RES in humans remains a challenge, and future research should focus on enhancing its absorption and developing more potent derivatives to maximize its anticancer effects (Bishayee 2009; Levi et al. 2005).

Based on the current study, RES shows promising potential for clinical application in the prevention and treatment of lung cancer. Here are some suggestions for its clinical use:

– Nanoparticle‐based delivery: RES‐loaded nanoparticles have demonstrated enhanced anticancer activity both in vitro and in vivo. This approach could improve resveratrol's bioavailability, stability, and targeted delivery to lung cancer cells (Annaji et al. 2021).
– Combination therapy: RES could be used in combination with standard chemotherapeutic agents to sensitize cancer cells and potentially reduce the required dosage of conventional drugs, thereby minimizing side effects (Ko et al. 2017).
– High‐risk individuals: RES supplementation could be considered for individuals at high risk of developing lung cancer, such as heavy smokers or those with a family history of the disease.
– Combination studies: Clinical trials evaluating RES in combination with standard lung cancer treatments should be conducted to assess potential synergistic effects and optimal dosing regimens.
– Formulation optimization: Further research is needed to develop RES formulations with improved bioavailability and targeted delivery to the lungs.

By implementing these suggestions, researchers and clinicians can work towards harnessing the potential of RES as a chemopreventive and therapeutic agent for lung cancer. However, it is crucial to conduct thorough clinical trials to establish its efficacy and safety in human subjects before widespread clinical application.

CUR has shown promising effects in enhancing cancer therapy based on numerous clinical studies. It can increase the effectiveness of chemotherapy and radiotherapy while reducing their side effects, leading to improved patient survival time and quality of life. In colorectal cancer studies, CUR increased efficacy in the large intestine, reduced glutathione S‐transferase activity, and decreased prostaglandin E2 production. It also reduced the number and size of polyps without significant toxicity. For pancreatic cancer, CUR decreased lipid peroxidation and increased glutathione content in patients. In prostate cancer, it lowered serum levels of prostate‐specific antigen when combined with isoflavones and reduced urinary symptom severity. During radiation therapy for breast cancer, CUR helped prevent skin symptoms, reduce patient pain and suffering, improve quality of life, and minimize treatment delays or interruptions. Overall, CUR can regulate multiple signaling pathways and molecular targets in cancer cells. Its low cost, pharmacological safety, efficiency, and multifaceted effects make it a promising agent for cancer prevention and treatment. However, more research is still needed to optimize dosing, bioavailability, and treatment protocols for maximum therapeutic benefit in different cancer types and stages. Close monitoring and collaboration between oncologists and integrative medicine practitioners are recommended when incorporating CUR into cancer treatment plans (Mansouri et al. 2020).

Based on the systematic review of clinical studies on curcumin's effects in cancer prevention and treatment (Mansouri et al. 2020), here are some suggestions for its use in lung cancer:

CUR may enhance the efficacy of chemotherapy drugs used to treat lung cancer. It could potentially reduce chemotherapy side effects and improve quality of life when combined with standard lung cancer treatments. Its supplementation during radiotherapy for lung cancer may help prevent and reduce the severity of radiation‐induced side effects. CUR has low bioavailability, so specialized formulations like nanoparticles or liposomal CUR may be needed to achieve therapeutic levels in lung tissue. While promising, curcumin's clinical applications in lung cancer care require further research to optimize dosing, formulations, and treatment protocols. Its use should be guided by individual patient factors and in consultation with the oncology care team.

### 9.3 Mechanism of Action Studies

Further research is needed to elucidate the precise mechanisms by which CUR and RES exert their anticancer effects. Future studies should:

– Investigate the interaction between CUR, RES, and other components of the STAT3 signaling pathway.
– Explore potential synergistic effects of combining CUR and RES with other natural compounds or conventional therapies.
– Examine the impact of these compounds on the tumor microenvironment, including their effects on tumor‐associated macrophages and angiogenesis.

### 9.4 Challenges With Using Curcumin and Resveratrol to Target STAT3 in Cancer Therapy

There are several important challenges:

– Nonspecific effects: These compounds can interact with multiple cellular targets beyond just STAT3, which may lead to off‐target effects or reduced specificity in cancer treatment.
– Variability in cellular responses: The effects of CUR and RES can vary significantly between different cancer cell types and even within the same cancer type. This heterogeneity makes it challenging to predict and standardize treatment outcomes.
– Formulation issues: Developing effective drug delivery systems to overcome the bioavailability and stability issues of these compounds remains a significant challenge.

Addressing these challenges through advanced drug delivery systems, chemical modifications, and well‐designed clinical trials is crucial for harnessing the full potential of CUR and RES in targeting STAT3 for cancer therapy.

### 9.5 Personalized Medicine Approaches

As the field of oncology moves towards more personalized treatment strategies, future research should:

– Identify biomarkers that can predict patient response to CUR and RES treatment.
– Investigate how genetic variations may influence the efficacy of these compounds in different patient populations.
– Develop tailored dosing regimens based on individual patient characteristics and tumor profiles.

### 9.6 Novel Analogues and Derivatives

Building on the promising results of CUR and RES analogues, future research should:

– Continue to develop and test novel analogues of CUR and RES with improved bioavailability and potency.
– Investigate the potential of combination therapies using different analogues to target multiple cancer pathways simultaneously.
– Explore the use of computational methods and artificial intelligence to design and screen new derivatives with enhanced anticancer properties.

By addressing these future directions, researchers can work toward translating the promising preclinical findings of CUR and RES into effective therapeutic strategies for lung cancer patients, potentially improving treatment outcomes and quality of life.

### 9.7 Side Effects

CUR and RES, while generally well‐tolerated, can produce adverse effects when consumed in high doses or for prolonged periods. CUR has been reported to cause gastrointestinal side effects such as nausea, diarrhea, and stomach upset in some individuals, and may also interfere with blood clotting and iron absorption (Liu et al. 2022; Asher and Spelman 2013).

RES supplementation has been associated with headaches, dizziness, and in rare cases, thrombocytopenia, highlighting the need for caution when using this compound as a dietary supplement (Hausenblas et al. 2015; Kumaran et al. 2018). Both compounds have shown potential to interact with certain medications and may affect liver enzyme levels, emphasizing the importance of consulting healthcare providers before initiating supplementation, especially for individuals with preexisting medical conditions or those taking other medications (Zhang et al. 2021; Detampel et al. 2012; Rivera‐Espinoza and Muriel 2009).

Consumption of these supplements can be beneficial when used under the supervision of healthcare specialists, who can monitor for potential side effects and drug interactions while tailoring dosages to individual needs.

## 10 Limitations

### 10.1 Scope of Study

This manuscript is a comprehensive review that synthesizes preclinical (in vitro *and* in vivo) and limited clinical data regarding CUR and RES effects on the STAT3 pathway in lung cancer. However, the majority of studies discussed are preclinical. Hence, direct clinical evidence supporting the efficacy and safety of CUR and RES as STAT3 modulators in lung cancer patients remains insufficient. Large‐scale, well‐designed clinical trials are necessary to validate these findings in humans.

### 10.2 Bioavailability Issues

Both CUR and RES are known to have poor bioavailability due to rapid metabolism and systemic elimination. Despite promising anticancer activities seen in experimental models, their therapeutic effectiveness in humans might be limited without improved drug delivery systems. Although nanoparticle‐based and hybrid formulations are emerging, more research is needed to optimize these delivery approaches and demonstrate their clinical relevance.

### 10.3 Heterogeneity of Lung Cancer

Lung cancer is a heterogeneous disease comprising subtypes like NSCLC and SCLC, each with distinct molecular profiles and treatment responses. The reviewed studies often do not differentiate the tumor heterogeneity sufficiently, which may influence the generalizability of STAT3‐targeted CUR and RES therapies across all lung cancer types.

### 10.4 Complexity of STAT3 Signaling

The STAT3 pathway interacts extensively with other signaling networks, and its role in cancer involves multiple downstream targets and feedback loops. Targeting STAT3 alone might not comprehensively suppress tumor progression due to compensatory mechanisms and pathway redundancies. Combination therapies with other agents may be required but were not extensively addressed in this review.

### 10.5 Dose and Treatment Duration Variability

There is considerable variability in the doses, treatment durations, and experimental conditions used across studies assessing CUR and RES, which complicates comparison and consensus building. Standardized protocols and dosing regimens are essential for translating preclinical results into clinical practice.

### 10.6 Potential Off‐Target Effects and Toxicity

While CUR and RES are generally considered safe in dietary amounts, their pharmacological use at higher doses may carry risks of off‐target effects and toxicity. The manuscript does not fully explore the safety profile and potential adverse effects when used as STAT3 inhibitors in lung cancer therapy.

## 11 Perspectives

### 11.1 Advancement of Clinical Trials

While preclinical studies provide strong evidence for the inhibitory effects of CUR and RES on STAT3 signaling and lung cancer progression, there is an urgent need for well‐designed clinical trials. Future clinical investigations should focus on establishing optimal dosing, formulations, and combination regimens of CUR and RES with current lung cancer therapies to validate their efficacy and safety in humans.

### 11.2 Improved Drug Delivery Systems

Given the low bioavailability and rapid metabolism of both CUR and RES, ongoing development of advanced nanoparticle‐based and hybrid delivery systems holds great promise. These systems could significantly enhance targeted delivery to lung tumor cells, improve pharmacokinetics, and overcome resistance mechanisms, ultimately enhancing therapeutic outcomes.

### 11.3 Combination Therapy Approaches

The complexity and redundancy of the STAT3 signaling network suggest that combining CUR and RES with other conventional treatments such as chemotherapy, targeted therapies, or immunotherapy may be essential. Investigating synergistic effects, reduced toxicity, and resistance reversal through combination strategies should be prioritized in future studies.

### 11.4 Subtype‐Specific Research

Considering the heterogeneity of lung cancer, further research should delineate the differential effects of CUR and RES on various lung cancer subtypes (NSCLC vs. SCLC) and molecular profiles. Precision medicine approaches integrating STAT3 modulation with genetic and epigenetic tumor markers could optimize patient stratification and treatment efficacy.

### 11.5 Targeting Tumor Microenvironment

The ability of resveratrol to modulate the tumor microenvironment, particularly by influencing tumor‐associated macrophages and immune responses, opens avenues for exploring immune modulation alongside STAT3 inhibition. Expanding focus on the interplay between STAT3 signaling, immune checkpoints, and inflammation could advance immunotherapeutic strategies.

### 11.6 Mechanistic Insights Into STAT3 Regulation

Deeper investigation into the molecular mechanisms by which CUR and RES regulate STAT3 phosphorylation and downstream gene expression, including cross‐talk with other signaling pathways, will enhance understanding of their full anticancer potential. Advanced molecular and omics approaches could identify novel biomarkers for therapy monitoring.

### 11.7 Addressing Drug Resistance

Since STAT3 is implicated in chemo‐ and immunotherapy resistance, future studies should explore the potential of CUR and RES to sensitize resistant lung cancer cells and reverse resistance pathways. This could contribute to extending patient survival and improving response rates.

### 11.8 Safety and Toxicological Evaluation

Comprehensive assessment of long‐term safety, pharmacodynamics, and potential off‐target effects of CUR and RES at therapeutic doses is essential before routine clinical use. Toxicology studies combined with pharmacovigilance in clinical settings will be crucial.

Implementing these perspectives will help translate the promising preclinical findings into tangible clinical benefits for lung cancer patients and pave the way for novel, effective adjunct therapies targeting STAT3.

## 12 Conclusion

This comprehensive review has elucidated the therapeutic potential of CUR and RES as dual modulators of the STAT3 pathway in lung cancer. The evidence presented demonstrates that both compounds exhibit potent anticancer effects through multiple mechanisms. CUR inhibits STAT3 activation, leading to reduced tumor growth, angiogenesis, and metastasis in nonsmall cell lung cancer (NSCLC) models. It downregulates the expression of STAT3, JAK2, VEGF, Bcl‐xL, and Cyclin D1, exhibiting significant antiangiogenic potency. Similarly, RES suppresses STAT3 signaling, inhibiting tumor growth, angiogenesis, and epithelial‐mesenchymal transition in NSCLC models. Notably, RES also inhibits the M2 polarization of tumor‐associated macrophages, disrupting the communication between tumor cells and the tumor microenvironment. The reviewed studies highlight the multifaceted approach of these natural compounds in targeting lung cancer. CUR analogues, such as L48H37 and compound 5 k, have shown promise in inhibiting cancer cell motility, migration, and invasion by modulating the JAK/STAT and NF‐κB/STAT3 signaling pathways. RES demonstrates potential in overcoming drug resistance in small cell lung cancer by inhibiting the STAT3/VEGF pathway and P‐glycoprotein function.

While these findings are promising, the translation of CUR and RES into clinical applications faces challenges, primarily due to their limited bioavailability. Future research should focus on developing advanced drug delivery systems, exploring novel analogues with enhanced bioavailability and potency, and conducting well‐designed clinical trials. The potential of combining these compounds with conventional therapies also warrants further investigation.

In conclusion, CUR and RES represent promising therapeutic agents for lung cancer management through their modulation of the STAT3 pathway. As research progresses, these natural compounds may play an increasingly important role in complementing existing treatment strategies, potentially improving outcomes for lung cancer patients.

## Author Contributions

**Mohammad Yasin Zamanian:** conceptualization (equal), investigation (equal), project administration (equal), supervision (equal), writing – review and editing (equal). **Klavdiya A. Turkadze:** writing – original draft (equal). **Mehraveh Sadeghi Ivraghi:** data curation (equal), methodology (equal), resources (equal), validation (equal). **Maryam Golmohammadi:** conceptualization (equal), writing – original draft (equal). **Maryam Sharifi:** data curation (equal), resources (equal), writing – original draft (equal). **Zahra Keshtpour Amlashi:** conceptualization (equal), project administration (equal), writing – review and editing (equal).

## Ethics Statement

The authors have nothing to report.

## Consent

The authors have nothing to report.

## Conflicts of Interest

The authors declare no conflicts of interest.

## Data Availability

The data used to support the findings of this study are available from the corresponding author upon request.
